# Polymorphisms in Radio-Responsive Genes and Its Association with Acute Toxicity among Head and Neck Cancer Patients

**DOI:** 10.1371/journal.pone.0089079

**Published:** 2014-03-04

**Authors:** Goutham Hassan Venkatesh, Vadhiraja Bejadi Manjunath, Kamalesh Dattaram Mumbrekar, Hitendra Negi, Donald Jerard Fernandes, Krishna Sharan, Sourjya Banerjee, Satish Rao Bola Sadashiva

**Affiliations:** 1 Division of Radiobiology & Toxicology, School of Life Sciences, Manipal University, Manipal, Karnataka, India; 2 Division of Biotechnology, School of Life Sciences, Manipal University, Manipal, Karnataka, India; 3 Manipal Hospital, Bangalore, Karnataka, India; 4 Department of Radiotherapy & Oncology, Kasturba Medical College and Hospital, Mangalore, Karnataka, India; 5 Department of Radiotherapy & Oncology, Shiridi SaiBaba Cancer Hospital and Research Centre, Kasturba Hospital, Manipal, Karnataka, India; University of California Davis, United States of America

## Abstract

Cellular and molecular approaches are being explored to find a biomarker which can predict the development of radiation induced acute toxicity prior to radiation therapy. SNPs in radiation responsive genes may be considered as an approach to develop tools for finding the inherited basis of clinical radiosensitivity. The current study attempts to screen single nucleotide polymorphisms/deletions in DNA damage response, DNA repair, profibrotic cytokine as well as antioxidant response genes and its predictive potential with the normal tissue adverse reactions from 183 head and neck cancer patients undergoing platinum based chemoradiotherapy or radiotherapy alone. We analysed 22 polymorphisms in 17 genes having functional relevance to radiation response. Radiation therapy induced oral mucositis and skin erythema was considered as end point for clinical radiosensitivity. Direct correlation of heterozygous and mutant alleles with acute reactions as well as haplotype correlation revealed NBN variants to be of predictive significance in analysing oral mucositis prior to radiotherapy. In addition, genetic linkage disequilibrium existed in XRCC1 polymorphisms for >grade 2 oral mucositis and skin reaction indicating the complex inheritance pattern. The current study indicates an association for polymorphism in NBN with normal tissue radiosensitivity and further warrants the replication of such studies in a large set of samples.

## Introduction

Precise radiation delivery methodologies have significantly improved tumor cure and survival rate owing to recent developments, however it has been at the expense of significant increase in normal tissue toxicity. Heterogeneity in normal tissue radioresponse is observed among patients treated with identical doses of radiation, which further leads to a dynamic and cumulative process of normal tissue toxicity. In head and neck cancer (HNC) patients, oral mucositis and skin erythema are the major complications during the course of chemoradiotherapy. It affects pain control and adequate treatment delivery, thereby leading to unanticipated radiotherapy (RT) breaks, compromising treatment efficacy [1]. Earlier clinical experiences have indicated that only 20% of the variability was because of stochastic or random events, whereas the rest 80% were because of patient related genetic factors [2]. The association between severe radiosensitivity and genetic syndromes like Ataxia-telangiectasia, Fanconi's anemia, and Bloom syndrome, etc., provides us a proof of the principle about the involvement of genetic component behind normal tissue acute reactions [3]. Also, previous findings demonstrate genotype-dependent cause for acute and late effects of RT seen in normal tissues [4] [5]. Apart from molecular aspects, our earlier studies suggest that there exist a cellular basis for normal tissue radiation sensitivity [6]. Two-third of the studies conducted till date report the association of genetic variation in candidate genes with radiation induced toxicity, but most of these studies are with small patient numbers and lacks independent validation [7]. Although studies have been conducted to associate the polymorphism in selected candidate genes with clinically observed normal tissue adverse effects, its clinical applicability as biomarker/s is still questionable [8] [9]. Therefore, well designed clinical studies with hundreds of samples are needed to seek a biomarker for developing individual treatment protocols [10] [11].

In the present study, single nucleotide polymorphisms/deletions in selected candidate genes related to DNA damage and repair, antioxidant response and detoxification enzymes and profibrotic cytokine were analysed. SNPs in candidate radiation responsive genes like ATM, XRCC1, XRCC3, XRCC4, Ku70, Ku80, LIG4, OGG1, NBN, RAD51, TGFβ1, SOD2, CAT and GST were selected. The severity of oral mucositis and skin reaction was recorded according to Radiation Therapy Oncology Group (RTOG) criteria [12] and the association between genetic polymorphism and oral mucositis and skin reaction was evaluated for the increased risk of developing these normal tissue adverse reactions.

## Materials and Methods

### Patients and clinical data

The study was conducted from 183 HNC patients undergoing chemoradiotherapy at Kasturba Hospital, with a prior approval by the University Ethical Committee (UEC/15/2007) and a written informed consent from the patients before collecting blood prior to RT. All patients were treated using 3-Dimensional Conformal Radiotherapy. Gross tumor volume (GTV), Clinical Target Volume (CTV) and Planning target volume (PTV) were defined by using these planning CT scan. Gross tumor volume encompassed all known gross disease as defined by clinical physical examination and imaging findings. Patients with gross disease were treated using Linac 6-MV X-ray linear accelerator (Elekta Precise digital, Stockholm, Sweden) with the total tumor dose of 70 Gy (2 Gy per day for 5 days week). Patients after surgical resection having positive margins were given a dose of 66 Gy in 33 fractions. Patients with no positive margins were given 60 Gy in 30 fractions. Dose to parotid gland, submandibular salivary glands, constrictor muscles and other structures were not restricted in view of 3-Dimensional treatment planning in all the patients. Cisplatin chemotherapy (100 mg/m^2^ for once in 3 weeks) was given to majority of the patients when serum creatinine was normal. Elderly patients received a weekly dose of 40 mg/m^2^ for 6 weeks and patients having borderline elevation of serum creatinine received carboplatin (area under curve (AUC) @ 1.5) on a weekly basis for 6 weeks. A total of 148 patients received concurrent chemoradiotherapy and the remaining patients were received radiotherapy alone. Patients with recurrent tumour and distant metastasis were excluded. Acute adverse events (oral mucositis and skin reaction) were recorded during and after completion of therapy according to RTOG criteria [10]. The details of HNC patient characteristics are described in table 1.

**Table 1:** Demographic and clinical details of head and neck cancer patients.

Patient clinical details		
Number of patients		183
Mean age		55 (26–80)
Males		157
Females		26
Smoking/Tobacco chewing		122
Alcohol consumption		66
Region	Hypopharynx	32
	Oropharynx	85
	Nasopharynx	5
	Larynx	27
	Oral cavity	15
	Para nasal region	11
	Parotid	4
	Thyroid	3
	Unknown origin of region	1
Tumor staging	T1	9
	T2	32
	T3	48
	T4	65
	Tx	12
Treatment	Chemoradiotherapy	148
	RT alone	35
Skin reaction (RTOG Grading)	Grade 0	3
	Grade I	27
	Grade II	97
	Grade III	36
	Grade IV	3
Mucositis (RTOG Grading)	Grade 0	1
	Grade I	8
	Grade II	57
	Grade III	48
	Grade IV	6

### Genotyping, haplotype and linkage disequilibrium analysis

Genomic DNA isolation was performed by employing the conventional phenol chloroform extraction and ethanol precipitation procedure. Genotyping was performed by Polymerase Chain Reaction based Restriction Fragment Length Polymorphism. The details are available in table 2. Five percent of the samples were randomly selected and re-genotyped to assess the consistency in results.

**Table 2:** The list of candidate genes selected in the present study.

Gene	rs number	Amino acid/nucleotide change	Chromosome	PCR product size (bp)	Enzyme	Dominant (wild type)	Heterozygous	Recessive (mutant)
***DNA damage and repair genes***								
*XRCC1*	rs25487	Gln399Arg (A>G)	Chr 19	615	*Msp I*	615	615, 377, 238	377, 238
*XRCC1*	rs1799782	Arg194Trp (C>T)	Chr 19	491	*Msp I*	292,178,21	313, 292, 178, 21	313, 178
*XRCC1*	rs25489	Arg280His (G>A)	Chr 19	280	*Rsa I*	280	280, 140	140
*XRCC1*	rs3213245	−77 (C>T)	Chr 19	219	*BsrB I*	173, 46	173,116, 57, 46	116, 57, 46
*XRCC3*	rs861539	Thr241Met (C>T)	Chr 14	336	*Nla III*	336	336, 231, 105	231, 105
*XRCC4*	rs1805377	894–7 (A>G)	Chr 5	170	*Tsp509 I*	170	170, 88, 82	88, 82
*XRCC5 (Ku80)*	rs3835	2110–2408 (G>A)	Chr 2	151	*Alu I*	78, 73	151,78,73	151
*XRCC6 (Ku70)*	rs2267437	88+57 (C>G)	Chr 22	178	*Nar I*	178	178,147, 31	147,31
*LIG4*	rs1805388	Thr9Ile, 26C>T	Chr 13	121	*HpyCH4 III*	65, 56	121, 65, 56	121
*NBN*	rs1805794	Glu185Gln, 553G>C	Chr 8	174	*Hinf I*	125, 49	174, 125, 49	174
*NBN*	rs1805787	1125–520 G>C	Chr 8	197	*Ear I*	172, 25	197, 172, 25	197
*RAD51*	rs1801320	−98G>C	Chr 19	131	*BstN I*	71, 60	131, 71, 60	131
*RAD51*	rs1801321	−61G>T	Chr 19	131	*NgoM IV*	110, 21	131, 110, 21	131
*ATM*	rs3218698	3285-10delT	Chr 11	200	*Fnu4H I*	200	200, 176, 24	176,24
*OGG1*	rs1052133	Ser326Cys (C>G)	Chr 3	156	*Fnu4HI*	156	156, 100, 56	100, 56
***Profibrotic and inflammatory cytokine***								
*TGF-β1*	rs1800469	509C>T	Chr 19	418	*Bsu36I*	229,189	418,229,189	418
***Antioxidant genes***								
*CAT*	rs7943316	−21A>T	Chr 11	250	*Hinf I*	177, 73	250, 177, 73	250
*SOD2*	rs4880	Val16Ala (C>T)	Chr 6	207	*BsaWI*	207	207, 167, 40	167, 40
*NQO1*	rs1131341	C>T	Chr 16	194	*Msp I*	102,92	194,102,94	194
***Detoxification genes***								
*GSTP1*	rs1695	Ile105Val (G>A)	Chr 11	433	*BsmAI*	222,106,105	328,222,106,105	328, 106, 105
*GSTT1*	-	Present/Null	Chr 22	480				
*GSTM1*	-	Present/Null	Chr 1	240				

### Statistical analysis

Each polymorphism was tested for deviation from Hardy-Weinberg equilibrium. Statistical significance was analysed by Fisher exact test. Odds ratio was estimated to test whether any association exist between the grade of acute toxicity and selected SNP/haplotypes. Haplotype analysis and linkage disequilibrium estimates were done using SHEsis software [13]. All statistical tests were performed using Prism v.5.0 (GraphPad Software, San Diego, California, USA) and Statistical Package for Social Science (Version 16.0, Chicago, USA).

## Results

The distribution of patients based on histopathological grading, tumor stage and acute toxicity grades is provided in table 1. The mean age group of the patients considered in the study was 54.74 years. Radiation doses ranging from 60 to 70 Gy (median = 66 Gy) in 30 to 35 fractions were given to the patients. A total of 148 patients underwent platinum-based chemoradiotherapy, while the remaining was given radiotherapy alone. Out of 183 patients, 71 (38.79%) patients experienced severe mucositis (grade 3 and 4) and 44 (24.04%) experienced severe skin reactions. We analysed the presence of confounding factors like diabetes, hypertension, smoking, alcohol, surgery and chemotherapy and found that patients with alcoholism was associated with grade ≤2 mucositis (p = 0.02) (Table 3).

**Table 3:** Effect of confounding factors on the set of samples analysed.

Confounding factors	Grade ≤2 Skin reaction (n = 139)	Grade >2 Skin reaction (n = 44)	p-value	Grade ≤2 Mucositis (n = 66)	Grade >2 Mucositis (n = 54)	p-value
**Age**	54.55±11.041	54.41±13.191	0.948	51.98±11.48	54.15±12.49	0.326
**Gender**	120m, 19f	37m, 7f	0.805	57m, 9f	45m, 9f	0.798
**Diabetes**	19	4	0.305	7	7	0.778
**Hypertension**	14	9	0.114	7	8	0.583
**Alcohol**	53	13	0.369	29	12	0.020*
**Smoking**	93	29	1.000	47	35	0.555
**Surgery**	51	15	0.858	27	18	0.451
**Chemotherapy**	111	37	0.148	52	47	0.429

According to radiation oncologists, toxicity upto grade 2 is usually tolerated by patients without any therapeutic intervention but grade 3 and 4 requires intervention with therapeutic agents. Based on this, and considering reports from earlier studies [14] [15], we grouped the patient normal tissue toxicity data as grade ≤2 or >2. Since the dose exposed to oral cavity/pharyngeal region varies from 0–40% in hypopharynx, larynx, thyroid and region of unknown origin, data from cancers in these regions were excluded for analysing oral mucositis. Of all the polymorphisms tested, RAD51 (rs1801321) Ku70 (rs2267437) and XRCC4 (rs1805377) were found to deviate from Hardy-Weinberg equilibrium. Univariate analysis showed that none of the polymorphisms presented any significant association to skin reaction (Table 4). However, it indicated that odds of patients experiencing severe oral mucositis (grade >2) with recessive allele of NBN (rs1805794) was 3.75 times higher having a confidence interval of 1.201–11.70 and p = 0.023. Also, heterozygous variants in CAT (rs7943316) displayed 0.452 (odds value) times lesser prone to experience severe oral mucositis (grade >2) with a confidence interval of 0.206–0.993 and p = 0.048. In continuation, multivariate analysis indicated that odds of patients experiencing severe oral mucositis (grade >2) with recessive allele of NBN (rs1805794) was 4.72 times higher having a confidence interval of 1.384–16.151 and p = 0.013 (Table 5). However, when we categorized the data as chemoradiotherapy and radiotherapy as separate groups, we did not observe any such significant association with normal tissue toxicity in chemoradiotherapy group (Table S1 and Table S2). Further, as the sample number is less in radiation therapy alone (n = 35) it is difficult to effectively conclude the findings.

**Table 4:** Univariate analysis of candidate single nucleotide polymorphisms and radiation-induced oral mucositis and skin reactions in head and neck cancer patients.

Gene name	Genotype	Skin reaction ≤2 (n = 139)	Skin reaction >2 (n = 44)	Odds ratio	95% CI	p-value	Oral Mucositis ≤2 (N = 66)	Oral Mucositis >2 (N = 54)	Odds ratio	95% CI	p-value
**XRCC1 (rs25487)**	AA	50	17	Reference			29	23	Reference		
	GA	62	14	0.411	0.154–1.096	0.076	28	25	1.339	0.418–4.295	0.623
	GG	15	8	0.545	0.204–1.458	0.227	9	6	1.190	0.370–3.829	0.771
**XRCC1 (rs1799782)**	CC	95	31	Reference			48	40	Reference		
	CT	30	6	0.624	0.253–1.537	0.305	15	12	.960	.403–2.285	0.927
	TT	2	2	1.962	0.315–12.228	0.470	3	2	.800	.127–5.026	0.812
**XRCC1 (rs25489)**	AA	97	30	Reference			52	41	Reference		
	GA	30	8	0.727	0.307–1.722	0.469	14	13	1.087	.454–2.603	0.851
	GG	0	1	3.000	0.183–49.239	0.442	0	0	2.049	000	1.000
**XRCC1 (rs3213245)**	TT	58	21	Reference			28	27	Reference		
	TC	53	16	0.955	0.469–1.943	0.898	27	23	0.883	0.410–1.903	0.751
	CC	16	2	0.452	0.122–1.677	0.235	11	4	0.377	0.107–1.330	0.129
**RAD51 (rs1801321)** *	GG	81	20	Reference			45	30	Reference		
	GT	21	11	1.891	0.842–4.247	0.123	12	14	1.750	0.712–4.299	0.222
	TT	25	8	1.164	0.466–2.909	0.745	9	10	1.667	0.606–4.586	0.323
**RAD51 (rs1801320)**	GG	90	29	Reference			48	35	Reference		
	CG	34	9	.842	0.377–1.880	0.674	17	17	1.371	0.616–3.055	0.440
	CC	3	1	1.010	0.102–10.04	0.993	1	2	2.743	0.239–31.45	0.418
**NBN (rs1805794)**	GG	44	13	Reference			24	16	Reference		
	CG	62	13	0.906	0.409–2.010	0.808	36	23	0.958	0.422–2.178	0.919
	CC	21	13	2.110	0.853–5.223	0.106	6	15	3.750	1.201–11.70	**0.023**
**NBN (rs1805787)**	GG	87	30	Reference			44	40	Reference		
	GC	36	8	0.599	0.254–1.411	0.241	22	12	0.550	0.237–1.275	0.164
	CC	4	1	0.554	0.063–4.911	0.596	0	2	1.777E9	.000	0.999
**OGG1 (rs1052133)**	CC	57	13	Reference			31	26	Reference		
	CG	53	19	1.235	0.586–2.605	0.579	25	23	1.097	0.508–2.368	0.814
	GG	17	7	1.560	0.583–4.176	0.376	10	5	0.596	0.181–1.955	0.396
**GSTP1 (rs1695)**	AA	63	21	Reference			37	30	Reference		
	AG	52	14	0.737	0.355–1.528	0.412	25	18	0.888	0.410–1.925	0.764
	GG	12	4	0.933	0.275–3.162	0.912	4	6	1.850	0.478–7.163	0.373
**GSTM1**	1	88	28	Reference			47	33	Reference		
	0	39	11	0.866	0.422–1.778	0.695	19	21	0.635	0.296–1.363	0.244
**GSTT1**	1	100	32	Reference			48	46	Reference		
	0	27	7	1.394	0.563–3.446	0.473	18	8	2.156	0.854–5.442	0.104
**CAT (rs7943316)**	TT	55	14	Reference			21	25	Reference		
	TA	62	17	0.532	0.532–2.356	0.766	39	21	0.452	0.206–0.993	**0.048**
	AA	10	8	0.874	0.874–7.151	0.087	6	8	1.120	0.335–3.745	0.854
**ATM (rs3218698)**	T/T	113	36	Reference			56	49	Reference		
	T/−T	14	3	0.718	0.228–2.258	0.571	10	5	0.571	0.183–1.787	.336
**Ku80 (rs3835)**	AA	96	27	Reference			53	39	Reference		
	GA	25	12	1.543	0.701–3.400	0.282	11	14	1.730	0.709–4.218	0.228
	GG	6	0	0.000	0.000 -	0.999	2	1	0.679	0.059–7.763	0.756
**KU70 (rs2267437)** *	CC	82	27	Reference			41	37	Reference		
	CG	33	10	0.812	0.370–1.784	0.605	19	13	0.758	0.329–1.745	0.515
	GG	12	2	0.401	0.086–1.865	0.244	6	4	0.739	0.193–2.824	0.658
**XRCC4 (rs1805377)** *	GG	94	28	Reference			44	40	Reference		
	GA	28	9	1.028	0.455–2.325	0.947	20	11	0.605	0.258–1.417	0.247
	AA	5	2	1.062	0.204–5.526	0.943	2	3	1.650	0.262–10.386	0.594
**XRCC3 (rs861539)**	CC	87	29	Reference			42	32	Reference		
	CT	46	15	0.978	0.477–2.007	0.952	22	22	1.313	0.621–2.775	0.477
	TT	6	0	0.000	0.000	0.999	2	0	0.000	0.000	0.999
**LIG4 (rs1805388)**	CC	100	32	Reference			52	48	Reference		
	CT	26	6	0.724	0.292–1.794	0.486	13	4	0.333	0.102–1.093	0.070
	TT	1	1	1.500	0.132–17.03	0.744	1	2	2.167	0.190–24.66	0.533
**SOD2 (rs4880)**	CC	29	11	Reference			15	18	Reference		
	CT	71	17	0.688	0.307–1.542	0.364	35	24	0.571	0.242–1.350	0.202
	TT	27	11	0.992	0.386–2.548	0.987	16	12	0.625	0.227–1.724	0.364
**TGF β1(rs1800469)**	CC	62	16	Reference			30	23	Reference		
	CT	50	16	1.361	0.646–2.869	0.417	29	23	1.034	0.478–2.237	0.931
	TT	15	7	1.855	0.686–5.015	0.224	7	8	1.491	0.472–4.711	0.496
**NQO1 (rs1131341)**	CC	103	33	Reference			55	45	Reference		
	CT	21	6	0.818	0.309–2.168	0.687	9	8	1.086	0.388–3.045	0.875
	TT	3	0	0.000	0.000	0.999	2	1	0.611	0.054–6.959	0.692

* Not in HWE.

**Table 5:** Multivariate analysis for CAT and NBN polymorphisms with radiation-induced oral mucositis in presence of alcohol among head and neck cancer patients.

Gene name	Genotype	Oral mucositis ≤2 (n = 66)	Oral mucositis >2 (n = 54)	Adjusted Odds ratio	95% CI	p- value
***CAT*** ** (rs7943316)**	TT	21	25	Reference		
	TA	39	21	0.463	0.199–1.076	0.074
	AA	6	8	1.675	0.427–6.575	0.460
***NBN*** ** (rs1805794)**	GG	24	16	Reference		
	CG	36	23	1.275	0.531–3.062	0.587
	CC	6	15	4.728	1.384–16.151	**0.013**

### Haplotyping and combination of risk alleles

**Figure 1:**
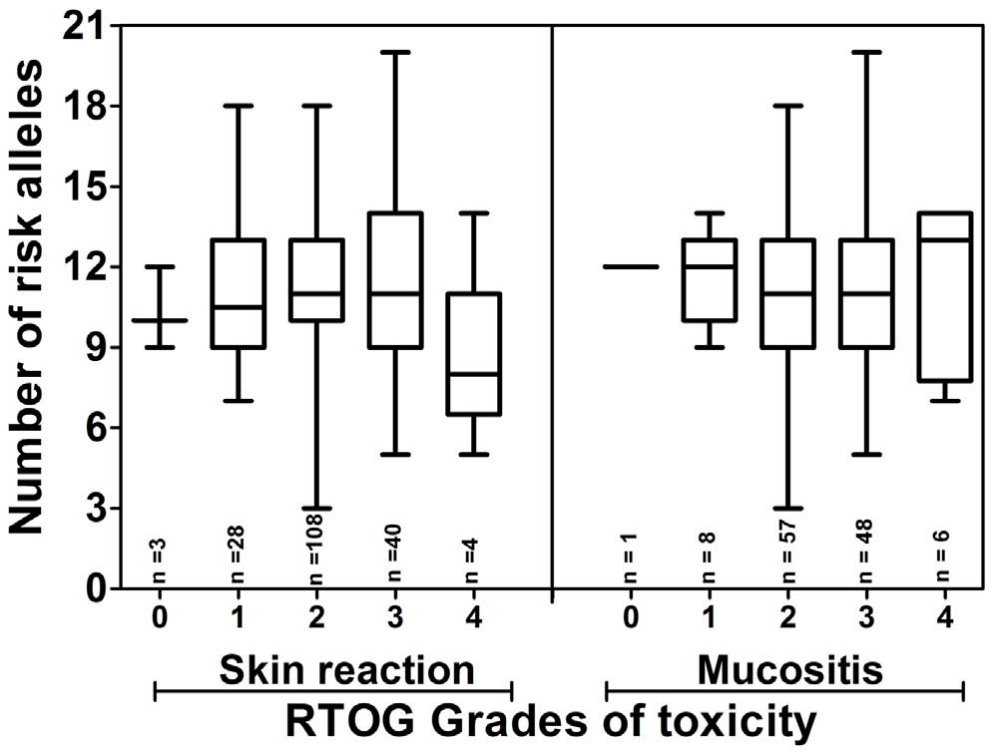
Association of average number of risk allele with the increasing RTOG grades of normal tissue toxicity. The results of Kruskal-Wallis test demonstrate that the comparison between the groups are non-significant (p>0.05). The error bars represent the minimum to maximum values of risk allele represented in each group.

Haplotype analysis was done for 4 SNPs in XRCC1, 2 polymorphisms in RAD51 as well as 2 polymorphisms in NBN to explore association of the combinatorial effect of these variants with increased normal tissue radiosensitivity. Haplotype analysis of NBN (rs1805787, rs1805794) gene demonstrated G-C haplotype to be associated with development of oral mucositis (odds ratio of 1.687 and 95% CI of 1.005–2.831 with p = 0.047) (Table 6). Further, to determine if any multiple SNP has an additive effect on the oral mucositis as well as skin reaction, the average number of variant alleles per patient in each RTOG group was analysed (Figure 1). Based on the allele frequency reported in the dbSNP database, we have reported the wild type, heterozygous and recessive genotypes. We considered the minor allele in this group as the risk allele and counted the number of risk alleles in each patient. The results suggest that the number of variant alleles has no effect on severity of normal tissue toxicity.

**Table 6:** Haplotype analysis for XRCC1 (rs3213245, rs1799782, rs25489 and rs25487), *RAD51* (rs1801320, rs1801321) and *NBN* (rs1805787, rs1805794) and radiation-induced oral mucositis and skin reactions in head and neck cancer patients.

Gene	Haplotype	Skin reactions				Oral mucositis			
		OR frequency	NOR frequency	Fisher's p-value	Odds ratio (95% CI)	OR frequency	NOR frequency	Fisher's p-value	Odds ratio (95% CI)
***XRCC1***	C-C-A-A	20.00 (0.256)	72.86 (0.287)	0.406	0.782 (0.438–1.397)				
	C-C-A-G	-	-	-	-	28.05 (0.260)	43.53 (0.330)	0.195	0.687 (0.389–1.215)
	T-C-A-A	9.07 (0.116)	33.72 (0.133)	0.573	0.799 (0.365–1.747)	32.20 (0.298)	39.81 (0.302)	0.873	0.955 (0.544–1.676)
	T-C-A-G	28.93 (0.371)	76.49 (0.301)	0.414	1.250 (0.731–2.136)	16.05(0.149)	12.94 (0.098)	0.252	1.576 (0.719–3.453)
	T-C-G-A	10.0 (0.128)	25.78 (0.101)	0.627	1.213 (0.556–2.648)	-	-	-	-
	T-C-G-G	-	-	-	-	12.75 (0.118)	9.71 (0.074)	0.258	1.654 (0.687–3.986)
	T-T-A-A	8.93 (0.114)	26.28 (0.103)	0.918	1.043 (0.465–2.338)				
	T-T-A-G	-	-	-	-	13.90 (0.129)	17.38 (0.132)	0.897	0.951 (0.445–2.033)
***RAD***	G-C	7.97 (0.102)	32.60 (0.128)	0.537	0.773 (0.340–1.754)	16.65 (0.154)	16.70 (0.127)	0.538	1.258 (0.605∼2.616)
	G-G	43.03 (0.552)	150.40 (0.592)	0.526	0.848 (0.508–1.414)	57.35 (0.531)	85.30 (0.646)	0.070	0.620 (0.369∼1.043)
	T-C	3.03 (0.039)	7.40 (0.029)	0.667	1.347 (0.345–5.258)	4.35 (0.040)	2.30 (0.017)	0.282	2.371 (0.470∼11.968)
	T-G	23.97 (0.307)	63.60 (0.250)	0.319	1.328 (0.760–2.322)	29.65 0.275)	27.70 (0.210)	0.242	1.425 (0.786∼2.583)
***NBN***	C-G	39.0 (0.500)	103.99 (0.409)	0.158	1.442 (0.867–2.401)	16.00 (0.148)	21.99 (0.167)	0.696	0.870 (0.431∼1.753)
	G-C	10.0 (0.128)	43.99 (0.173)	0.346	0.702 (0.335–1.470)	53.00 (0.491)	47.99 (0.364)	**0.047**	1.687 (1.005∼2.831)
	G-G	29.0(0.372)	106.01 (0.417)	0.473	0.826 (0.490–1.393)	39.00 (0.361)	62.01 (0.470)	0.089	0.638 (0.379∼1.074)

All those haplotypes with frequency <0.03 were ignored during the analysis.

**Figure 2:**
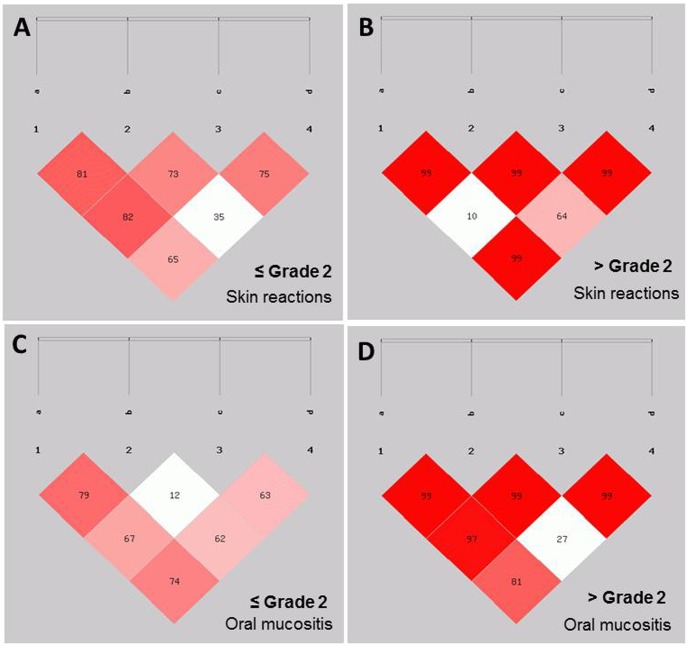
Linkage disequilibrium analysis for XRCC1 polymorphisms (a = rs3213245, b = rs1799782, c = rs25487, d = rs25489) for skin reaction (A and B) and oral mucositis (C and D). The numbers inside every box represent r2 values (%) of the linkage disequilibrium.

Furthermore, we analysed the linkage disequilibrium pattern for SNPs in XRCC1 and found that rs3213245, rs1799782, rs25489 and rs25487 were linked with severe oral mucositis while, rs1799782 and rs25489 were linked with severe skin reaction. Also, to validate this linked SNPs were analysed for linkage disequilibrium pattern separately in normal tissue overreactor and non-overreactor phenotypes. The d′ values were strikingly higher in normal tissue radiosensitive phenotypes (Figure 2).

## Discussion

Although, the growing volume of SNP data suggests the genetic basis for susceptibility to radiotherapy induced acute effects, it is less clear whether the SNPs can serve as a biomarker for predicting the normal tissue toxicity. Identifying the genetic profiles associated with an enhanced or reduced risk for radiotherapy complications seems to be a most promising factor to improve the efficacy of radiotherapy [9] [16]. The amount of human genetic diversity is immense, and we are just now beginning to understand how such changes influence the specific phenotypic expression. Remarkable genetic variations exist among populations and understanding this variation will help us to tailor the therapy with a personalised approach for safer and effective outcome.

Based on the functional significance of genes in radiation response, we analysed the association of some of the important gene variants belonging to DNA damage response, DNA repair, profibrotic cytokine, antioxidant genes with normal tissue overreactor phenotype (grade >2 toxicity). We did not find any significant association for either the selected SNPs or for the haplotypes with the risk of developing oral mucositis and skin reaction in HNC patients. However, we observed an association for NBN (rs1805794) polymorphism in univariate as well as multivariate model of analysis. In addition, one of the NBN haplotype was associated with severe oral mucositis. NBN is a component of MRE complex (MRE11-RAD50-NBN) which is involved in damage sensing, signaling and responding to DSBs [17]. This polymorphism brings about the change in Nibrin protein at 185th codon position from glutamic acid to glutamine and the functional significance of this change still remains unclear [18]. It was reported that rs1805794 was not associated with acute side effects of radiotherapy in breast cancer patients [18] [19]. Also, there are studies which report no association for rs1805794 and late radiation toxicities [20] [21].

Currently there are only few studies related to normal tissue toxicity and genotype analysis in HNC patients, and it is less clear whether the SNPs can serve as a biomarker for predicitng the normal tissue toxicity. Werbrouck and co-workers [22] report that SNPs in DNA repair genes XRCC3 (rs861539) and Ku70 (rs2267437) may help in determining the risk for acute dysphagia. Study conducted by Pratesi et al. [23] has suggested that patients with XRCC1 (rs25487) and RAD51 (rs1801320) have higher likelihood of developing oral mucositis and dysphagia in HNC patients. Unlike previous reports, null variants of GSTM1 and GSTT1 also showed no association with the development of acute reactions [24]. Also, a large-scale analysis for screening 3,144 SNPs from 156 breast cancer patients has revealed that ABCA1 and IL12RB2 polymorphism are highly susceptible to radiation-induced dermatitis [25]. Conversely, several studies aimed at validating the effect of TGFβ1 [26], [27], ATM, GSTP1, SOD2, TGFβ1, XPD and XRCC1 [28] have indicated no such association for the risk of developing normal tissue toxicity. Earlier reports with the approach of haplotype analysis have revealed several haplotypes with significant evidence for predicting acute reactions of radiotherapy [4] [29] [30]. Our analysis for XRCC1 haplotype did not associate with the risk of increased acute reactions, but NBN haplotype had an association for oral mucositis.. Studies suggest that SNPs or haplotypes in functionally important candidate genes alone may not contribute to radiation induced acute reactions. In addition, extensive literature review [8], [31] has indicated a contradictory association for susceptibility to radiation induced toxicity, which supports the need for further studies.

In conclusion, we report that gene variants and haplotypes of NBN are associated with the risk of developing oral mucositis in head and neck cancer patients undergoing chemoradiotherapy/radiotherapy. In addition to the screening for rare variants having large effects and common variants with small effects, we need to identify other types of variation and explore gene environment interactions for developing predictive models. Further replication of our results in large data sets with pathway-based approach and large genome wide association studies with methodological approach can be of great use to develop predictive biomarkers for this complex trait and the findings must be independently confirmed in different populations.

## Supporting Information

Table S1 **Univariate analysis of candidate single nucleotide polymorphisms and radiation-induced skin reactions after categorising the samples based on chemoradiotherapy and radiotherapy alone.**
(DOCX)

Table S2 **Univariate analysis of candidate single nucleotide polymorphisms and radiation-induced oral mucositis after categorising the samples based on chemoradiotherapy and radiotherapy alone.**
(DOCX)

## References

[1] Trotti A (2000) Toxicity in head and neck cancer: a review of trends and issues. Int J Radiat Oncol Biol Phys 47: 1–12. 10758302 10.1016/s0360-3016(99)00558-1

[2] Safwat A, Bentzen SM, Turesson I, Hendry JH (2002) Deterministic rather than stochastic factors explain most of the variation in the expression of skin telangiectasia after radiotherapy. Int J Radiat Oncol Biol Phys 52: 198–204. 11777639 10.1016/s0360-3016(01)02690-6

[3] Barnett GC, West CM, Dunning AM, Elliott RM, Coles CE, et al (2009) Normal tissue reactions to radiotherapy: towards tailoring treatment dose by genotype. Nat Rev Cancer 9: 134–142. 19148183 10.1038/nrc2587 PMC2670578

[4] Ishikawa A, Suga T, Shoji Y, Kato S, Ohno T, et al (2011) Genetic variants of NPAT-ATM and AURKA are associated with an early adverse reaction in the gastrointestinal tract of patients with cervical cancer treated with pelvic radiation therapy. Int J Radiat Oncol Biol Phys 81: 1144–52. 21050672 10.1016/j.ijrobp.2010.09.012

[5] Giotopoulos G, Symonds RP, Foweraker K, Griffin M, Peat I, et al (2007) The late radiotherapy normal tissue injury phenotypes of telangiectasia, fibrosis and atrophy in breast cancer patients have distinct genotype-dependent causes. Br J Cancer 96: 1001–1007. 17325707 10.1038/sj.bjc.6603637 PMC2360097

[6] Goutham HV, Mumbrekar KD, Vadhiraja BM, Fernandes DJ, Sharan K, et al (2012) DNA Double-strand break analysis by γ-H2AX Foci: a useful method for determining the overreactors to radiation-induced acute reactions among head-and-neck cancer patients. Int J Radiat Oncol Biol Phys 84: e607–12. 22836053 10.1016/j.ijrobp.2012.06.041

[7] Andreassen CA, Dikomey E, Parliament M, West CML (2012) Will SNPs be useful predictors of normal tissue radiosensitivity in the future? Radiother Oncol 105: 283–288. 23245645 10.1016/j.radonc.2012.11.003

[8] Popanda O, Marquardt JU, Chang-Claude J, Schmezer P (2009) Genetic variation in normal tissue toxicity induced by ionizing radiation. Mut Res 667: 58–69. 19022265 10.1016/j.mrfmmm.2008.10.014

[9] Barnett GC, Coles CE, Elliott RM, Baynes C, Luccarini C, et al (2012) Independent validation of genes and polymorphisms reported to be associated with radiation toxicity: a prospective analysis study. Lancet Oncol 13: 65–77. 22169268 10.1016/S1470-2045(11)70302-3

[10] Zackrisson B, Mercke C, Strander H, Wennerberg J, Cavallin-Ståhl E (2003) A systematic overview of radiation therapy effects in head and neck cancer. Acta Oncol 42: 443–61. 14596506 10.1080/02841860310014886

[11] Parliament MB, Murray D (2010) Single nucleotide polymorphisms of DNA repair genes as predictors of radioresponse. Semin Radiat Oncol 20: 232–40. 20832015 10.1016/j.semradonc.2010.05.003

[12] Cox JD, Stetz J, Pajak TF (1995) Toxicity criteria of the Radiation Therapy Oncology Group (RTOG) and the European Organization for Research and Treatment of Cancer (EORTC). Int J Radiat Oncol Biol Phys 31: 1341–1346. 7713792 10.1016/0360-3016(95)00060-C

[13] Li Z, Zhang Z, He Z, Tang W, Li T, et al (2009) A partition-ligation combinationsubdivision EM algorithm for haplotype inference with multiallelic markers: update of the SHEsis. Available: http://analysis.bio-x.cn Cell Res 19: 519–23. 19290020 10.1038/cr.2009.33

[14] Guerra JL, Gomez D, Wei Q, Liu Z, Wang LE, et al (2012) Association between single nucleotide polymorphisms of the transforming growth factor β1 gene and the risk of severe radiation esophagitis in patients with lung cancer. Radiother Oncol 105: 299–304. 23022171 10.1016/j.radonc.2012.08.014

[15] Bentzen SM (2006) Preventing or reducing late side effects of radiation therapy: radiobiology meets molecular pathology. Nat Rev Cancer 6: 702–13. 16929324 10.1038/nrc1950

[16] West CM, Barnett GC (2012) Genetics and genomics of radiotherapy toxicity: towards prediction. Genome Med 3: e52. 10.1186/gm268 PMC3238178 21861849

[17] Silva J, Teixeira AL, Lobo F, Maurício J, Medeiros R (2012) DNA repair system and prostate cancer progression: the role of NBS1 polymorphism (rs1805794). DNA Cell Biol 3: 1182–6. 10.1089/dna.2011.1562 22413803

[18] Popanda O, Tan XL, Ambrosone CB, Kropp S, Helmbold I, et al (2006) Genetic polymorphisms in the DNA double-strand break repair genes XRCC3, XRCC2, and NBS1 are not associated with acute side effects of radiotherapy in breast cancer patients. Cancer Epidemiol Biomarkers Prev 15: 1048–50. 16702393 10.1158/1055-9965.EPI-06-0046

[19] Millikan RC, Player JS, Decotret AR, Tse CK, Keku T (2012) Polymorphisms in DNA repair genes, medical exposure to ionizing radiation, and breast cancer risk. Cancer Epidemiol Biomarkers Prev 14: 2326–34. 10.1158/1055-9965.EPI-05-0186 16214912

[20] Damaraju S, Murray D, Dufour J, Carandang D, Myrehaug S, et al (2006) Association of DNA repair and steroid metabolism gene polymorphisms with clinical late toxicity in patients treated with conformal radiotherapy for prostate cancer. Clin Cancer Res 12: 2545–54. 16638864 10.1158/1078-0432.CCR-05-2703

[21] Chang-Claude J, Ambrosone CB, Lilla C, Kropp S, Helmbold I, et al (2009) Genetic polymorphisms in DNA repair and damage response genes and late normal tissue complications of radiotherapy for breast cancer. Br J Cancer 100: 1680–6. 19367277 10.1038/sj.bjc.6605036 PMC2696768

[22] Werbrouck J, De Ruyck K, Duprez F, Veldeman L, Claes K, et al (2009) Acute normal tissue reactions in head-and-neck cancer patients treated with IMRT: influence of dose and association with genetic polymorphisms in DNA DSB repair genes. Int J Radiat Oncol Biol Phys 73: 1187–1195. 19251090 10.1016/j.ijrobp.2008.08.073

[23] Pratesi N, Mangoni M, Mancini I, Paiar F, Simi L, et al (2011) Association between single nucleotide polymorphisms in the XRCC1 and RAD51 genes and clinical radiosensitivity in head and neck cancer. Radiother Oncol 99: 356–361. 21704413 10.1016/j.radonc.2011.05.062

[24] Ambrosone CB, Tian C, Ahn J, Kropp S, Helmbold I, et al (2006) Genetic predictors of acute toxicities related to radiation therapy following lumpectomy for breast cancer: a case-series study. Breast Can Res 8: R40. 10.1186/bcr1526 PMC1779469 16848913

[25] Isomura M, Oya N, Tachiiri S, Kaneyasu Y, Nishimura Y, et al (2008) IL12RB2 and ABCA1 genes are associated with susceptibility to radiation dermatitis. Clin Cancer Res 14: 6683–9. 18927311 10.1158/1078-0432.CCR-07-4389

[26] Voets AM, Oberije C, Struijk RB, Reymen B, De Ruyck K, et al (2012) No association between TGFβ1 polymorphisms and radiation-induced lung toxicity in a European cohort of lung cancer patients. Radiother Oncol 105: 296–8. 23127773 10.1016/j.radonc.2012.09.016

[27] Reuther S, Metzke E, Bonin M, Petersen C, Dikomey E, et al (2013) No effect of the transforming growth factor b1 promoter polymorphism c-509t on TGFβ1 gene expression, protein secretion, or cellular radiosensitivity. Int J Radiat Oncol Biol Phys 85: 460–65. 22592044 10.1016/j.ijrobp.2012.01.090

[28] Zschenker O, Raabe A, Boeckelmann IK, Borstelmann S, Szymczak S, et al (2010) Association of single nucleotide polymorphisms in ATM, GSTP1, SOD2, TGFβ1, XPD and XRCC1 with clinical and cellular radiosensitivity,. Radiother Oncol 97: 26–32. 20170971 10.1016/j.radonc.2010.01.016

[29] Suga T, Ishikawa A, Kohda M, Otsuka Y, Yamada S, et al (2007) Haplotype-based analysis of genes associated with risk of adverse skin reactions after radiotherapy in breast cancer patients. Int J Radiat Oncol Biol Phys 69: 685–93. 17889263 10.1016/j.ijrobp.2007.06.021

[30] Schirmer MA, Brockmöller J, Rave-Fränk M, Virsik P, Wilken B, et al (2011) A putatively functional haplotype in the gene encoding transforming growth factor beta- 1 as a potential biomarker for radiosensitivity. Int J Radiat Oncol Biol Phys 79: 866–74. 21183289 10.1016/j.ijrobp.2010.08.040

[31] Andreassen CN, Alsner J (2009) Genetic variants and normal tissue toxicity after radiotherapy: a systematic review. Radiother Oncol 92: 299–309. 19683821 10.1016/j.radonc.2009.06.015

