# Supplementary material for: Polymorphisms in Radio-Responsive Genes and Its Association with Acute Toxicity among Head and Neck Cancer Patients
Source: PLoS One. 2014 Mar 4;9(3):e89079. doi: 10.1371/journal.pone.0089079 (PMC3942321; doi:10.1371/journal.pone.0089079)
Supplement: Table S1 — Univariate analysis of candidate single nucleotide polymorphisms and radiation-induced skin reactions after categorising the samples based on chemoradiotherapy and radiotherapy alone. (DOCX) [file pone.0089079.s001.docx]

| **Gene** |  | **RT Alone (n = 35)** | | | | | | **Chemo RT (n = 148)** | | | | |
| --- | --- | --- | --- | --- | --- | --- | --- | --- | --- | --- | --- | --- |
|  | **Variants** | **Grade 2 skin reactions** | **Grade >2 skin reactions** | **Odds ratio** | **CI (95%)** | | **p-value** | **Grade 2 skin reactions** | **Grade >2 skin reactions** | **Odds ratio** | **CI (95%)** | **p-value** |
| **XRCC1**  **(rs25487)** | AA | 2 | 2 | Reference | | | | 13 | 7 | Reference | | |
|  | GA | 18 | 3 | 0.167 | 0.017- 1.679 | | 0.128 | 52 | 14 | 0.51 | 0.171 - 1.52 | 0.227 |
|  | GG | 8 | 2 | 0.25 | 0.021-3.041 | | 0.277 | 47 | 16 | 0.632 | 0.215 - 1.861 | 0.405 |
| **XRCC1 (rs1799782)** | CC | 21 | 6 | Reference | | | | 82 | 29 | Reference | | |
|  | CT | 7 | 0 | 0 | 0 - | | 0.999 | 26 | 7 | 0.761 | 0.299 - 1.941 | 0.568 |
|  | TT | 0 | 1 | 5.65E | 0 - | | 1 | 3 | 1 | 0.943 | 0.094 - 9.424 | 0.96 |
| **XRCC1**  **(rs25489)** | AA | 21 | 6 | Reference | | | | 84 | 29 | Reference | | |
|  | GA | 7 | 0 | 0 | 0 - | | 0.999 | 26 | 8 | 0.891 | 0.363 - 2.187 | 0.802 |
|  | GG | 0 | 1 | 5.65 | 0 - | | 1 | 1 | 0 | 0 | 0 - | 1 |
| **XRCC1 (rs3213245)** | TT | 12 | 5 | Reference | | | | 51 | 17 | Reference | | |
|  | TC | 12 | 2 | 0.4 | 0.065 - 2.48 | | 0.325 | 45 | 17 | 1.133 | 0.518 - 2.479 | 0.754 |
|  | CC | 4 | 0 | 0 | 0 - | | 0.999 | 15 | 3 | 0.6 | 0.155 - 2.328 | 0.46 |
| **OGG1 (rs1052133)** | CC | 9 | 4 | Reference | | | | 54 | 13 | Reference | | |
|  | CG | 13 | 3 | 0.519 | 0.093 - 2.904 | | 0.456 | 44 | 16 | 1.51 | 0.657 -3.475 | 0.332 |
|  | GG | 6 | 0 | 0 | 0 - | | 0.999 | 13 | 8 | 2.556 | 0.878 - 7.443 | 0.085 |
| **GSTP1**  **(rs1695)** | AA | 16 | 3 | Reference | | | | 54 | 22 | Reference | | |
|  | AG | 10 | 4 | 2.133 | 0.393 - 11.592 | | 0.38 | 47 | 11 | 0.574 | 0.252 - 1.308 | 0.187 |
|  | GG | 2 | 0 | 0 | 0 - | | 0.999 | 10 | 4 | 0.982 | 0.278 - 3.465 | 0.977 |
| **GSTM1** | present | 18 | 5 | Reference | | | | 78 | 24 | Reference | | |
|  | absent | 10 | 2 | 1.389 | 0.227 - 8.511 | | 0.722 | 33 | 13 | 0.781 | 0.355 - 1.718 | 0.539 |
| **GSTT1** | present | 23 | 5 | Reference | | | | 87 | 32 | Reference | | |
|  | absent | 5 | 2 | 0.543 | 0.081 - 3.647 | | 0.53 | 24 | 5 | 1.766 | 0.621 - 5.021 | 0.286 |
| **CAT (rs7943316)** | TT | 9 | 3 | Reference | | | | 51 | 13 | Reference | | |
|  | TA | 16 | 3 | 0.563 | 0.093 - 3.391 | | 0.53 | 51 | 17 | 1.308 | 0.576 - 2.969 | 0.521 |
|  | AA | 3 | 1 | 1 | 0.073 - 13.644 | | 1 | 9 | 7 | 3.051 | 0.956 - 9.737 | 0.06 |
| **TGF-**β**1 (rs1800469)** | CC | 16 | 4 | Reference | | | | 51 | 13 | Reference | | |
|  | CT | 8 | 3 | 1.5 | 0.268 - 8.383 | | 0.644 | 47 | 16 | 1.336 | 0.581 - 3.07 | 0.496 |
|  | TT | 4 | 0 | 0 | 0 - | | 0.999 | 13 | 8 | 2.414 | 0.827 - 7.044 | 0.107 |
| **NQO1**  **(rs1131341)** | CC | 21 | 6 | Reference | | | | 93 | 32 | Reference | | |
|  | CT | 7 | 1 | 0.5 | 0.051 - 4.904 | | 0.552 | 15 | 5 | 0.969 | 0.326 - 2.878 | 0.954 |
|  | TT | 0 | 0 |  |  | |  | 3 | 0 | 0 | 0 - | 0.999 |
| **ATM**  **(rs3218698)** | TT | 24 | 7 | Reference | | | | 98 | 33 | Reference | | |
|  | T/-T | 4 | 0 | 0 | 0 - | | 0.999 | 13 | 4 | 0.914 | 0.279 - 2.998 | 0.882 |
| **RAD51 (rs1801321)** | GG | 18 | 4 | Reference | | | | 69 | 19 | Reference | | |
|  | GT | 4 | 2 | 2.25 | 0.3 - 16.854 | | 0.43 | 22 | 11 | 1.816 | 0.75 - 4.396 | 0.186 |
|  | TT | 6 | 1 | 0.75 | 0.07 - 8.089 | | 0.813 | 20 | 7 | 1.271 | 0.468 - 3.453 | 0.638 |
| **RAD51**  **(rs1801320)** | GG | 19 | 6 | Reference | | | | 81 | 27 | Reference | | |
|  | CG | 9 | 1 | 0.352 | 0.037 - 3.374 | | 0.365 | 27 | 9 | 1 | 0.418 - 2.39 | 1 |
|  | CC | 0 | 0 |  |  |  |  | 3 | 1 | 1 | 0.1 - 10.021 | 1 |
| **NBN (rs1805794)** | GG | 9 | 2 | Reference | | | | 41 | 12 | Reference | | |
|  | CG | 14 | 2 | 0.643 | 0.076 - 5.417 | | 0.685 | 53 | 15 | 0.967 | 0.409 - 2.289 | 0.939 |
|  | CC | 5 | 3 | 2.7 | 0.332 - 21.977 | | 0.353 | 17 | 10 | 2.01 | 0.731 - 5.529 | 0.176 |
| **NBN**  **(rs1805787)** | GG | 21 | 5 | Reference | | | | 76 | 30 | Reference | | |
|  | GC | 6 | 1 | 0.7 | 0.068 - 7.201 | | 0.764 | 31 | 7 | 0.572 | 0.227 - 1.439 | 0.235 |
|  | CC | 1 | 1 | 4.2 | 0.222 - 79.319 | | 0.338 | 4 | 0 | 0 | 0 - | 0.999 |
| **Ku70**  **(rs2267437)** | CC | 22 | 5 | Reference | | | | 65 | 26 | Reference | | |
|  | CG | 5 | 2 | 1.76 | 0.262 - 11.835 | | 0.561 | 33 | 9 | 0.682 | 0.287 - 1.621 | 0.386 |
|  | GG | 1 | 0 | 0 | 0 - | | 1 | 13 | 2 | 0.385 | 0.081 - 1.824 | 0.229 |
| **Ku80**  **(rs3835)** | AA | 23 | 3 | Reference | | | | 84 | 29 | Reference | | |
|  | GA | 5 | 4 | 6.133 | 1.032 - 36.449 | | **0.046** | 21 | 8 | 1.103 | 0.441 - 2.761 | 0.833 |
|  | GG |  |  |  |  | |  | 6 | 0 | 0 | 0 - | 0.999 |
| **XRCC4**  **(rs1805377)** | GG | 23 | 7 | Reference | | | | 79 | 25 | Reference | | |
|  | GA | 5 | 0 | 0 | 0 - | | 0.999 | 26 | 10 | 1.215 | 0.516 - 2.863 | 0.655 |
|  | AA |  |  |  |  | |  | 6 | 2 | 1.053 | 0.2 - 5.553 | 0.951 |
| **LIG4**  **(rs1805388)** | CC | 24 | 5 | Reference | | | | 84 | 31 | Reference | | |
|  | CT | 4 | 2 | 2.4 | 0.341 - 16.899 | | 0.379 | 25 | 5 | 0.542 | 0.191 - 1.541 | 0.25 |
|  | TT |  |  |  |  | |  | 2 | 1 | 1.355 | 0.119 - 15.475 | 0.807 |
| **SOD2**  **(rs4880)** | CC | 8 | 3 | Reference | | | | 26 | 10 | Reference | | |
|  | CT | 17 | 2 | 0.314 | 0.043 - 2.265 | | 0.25 | 59 | 18 | 0.793 | 0.322 - 1.951 | 0.614 |
|  | TT | 3 | 2 | 1.778 | 0.192 - 16.492 | | 0.613 | 26 | 9 | 0.9 | 0.314 - 2.577 | 0.844 |
| **XRCC3 (rs861539)** | CC | 20 | 6 | Reference | | | | 67 | 23 | Reference | | |
|  | CT | 7 | 1 | 0.476 | 0.048 - 4.68 | | 0.525 | 39 | 14 | 1.046 | 0.483 - 2.265 | 0.91 |
|  | TT | 1 | 0 | 0 | 0 - | | 1 | 5 | 0 | 0 | 0 - | 0.999 |

Supplementary Table S1: Univariate analysis of candidate single nucleotide polymorphisms and radiation-induced skin reactions after categorising the samples based on chemoradiotherapy and radiotherapy alone.
