# Supplementary material for: Polymorphisms in Radio-Responsive Genes and Its Association with Acute Toxicity among Head and Neck Cancer Patients
Source: PLoS One. 2014 Mar 4;9(3):e89079. doi: 10.1371/journal.pone.0089079 (PMC3942321; doi:10.1371/journal.pone.0089079)
Supplement: Table S2 — Univariate analysis of candidate single nucleotide polymorphisms and radiation-induced oral mucositis after categorising the samples based on chemoradiotherapy and radiotherapy alone. (DOCX) [file pone.0089079.s002.docx]

| **Gene** |  | **RT Alone(n = 21)** | | | | | **Chemo RT (n = 99)** | | | | |
| --- | --- | --- | --- | --- | --- | --- | --- | --- | --- | --- | --- |
|  | **Variants** | **Grade ≤2 Mucositis** | **Grade >2 Mucositis** | **Odds ratio** | **CI (95%)** | **p-value** | **Grade ≤2 Mucositis** | **Grade >2 Mucositis** | **Odds ratio** | **CI (95%)** | **p-value** |
| **XRCC1**  **(rs25487)** | AA | 2 | 0 | Reference | | | 7 | 6 | Reference | | |
|  | GA | 10 | 4 | 6.46E | 0 - | 0.999 | 18 | 21 | 1.361 | 0.386- 4.794 | 0.631 |
|  | GG | 2 | 3 | 2.42E | 0 - | 0.999 | 27 | 20 | 0.864 | 0.252 - 2.969 | 0.817 |
| **XRCC1 (rs1799782)** | CC | 10 | 6 | Reference | | | 38 | 34 | Reference | | |
|  | CT | 4 | 0 | 0 | 0 | 0.999 | 11 | 12 | 1.219 | 0.476 - 3.121 | 0.679 |
|  | TT | 0 | 1 | 2.69E | 0 | 1 | 3 | 1 | 0.373 | 0.037 - 3.753 | 0.402 |
| **XRCC1**  **(rs25489)** | AA | 12 | 4 | Reference | | | 40 | 37 | Reference | | |
|  | GA | 2 | 3 | 4.5 | 0.542-37.378 | 0.164 | 12 | 9 | 0.811 | 0.306 - 2.145 | 0.673 |
|  | GG | 0 | 0 |  |  |  | 0 | 1 | 1.75E | 0 | 1 |
| **XRCC1 (rs3213245)** | TT | 6 | 3 | Reference | | | 22 | 24 | Reference | | |
|  | TC | 6 | 4 | 1.333 | 0.204 - 8.708 | 0.764 | 21 | 19 | 0.829 | 0.355 - 1.937 | 0.666 |
|  | CC | 2 | 0 | 0 | 0 - | 0.999 | 9 | 4 | 0.407 | 0.11 - 1.513 | 0.18 |
| **OGG1 (rs1052133)** | CC | 4 | 4 | Reference | | | 27 | 22 | Reference | | |
|  | CG | 7 | 3 | 0.429 | 0.062 - 2.972 | 0.391 | 18 | 20 | 1.364 | 0.583-3.19 | 0.474 |
|  | GG | 3 | 0 | 0 | 0 - | 0.999 | 7 | 5 | 0.877 | 0.244 - 3.147 | 0.84 |
| **GSTP1**  **(rs1695)** | AA | 9 | 2 | Reference | | | 28 | 28 | Reference | | |
|  | AG | 5 | 4 | 3.6 | 0.478 - 27.11 | 0.214 | 20 | 14 | 0.7 | 0.296 - 1.655 | 0.417 |
|  | GG | 0 | 1 | 7.27E | 0 - | 1 | 4 | 5 | 1.25 | 0.304 - 5.147 | 0.757 |
| **GSTM1** | present | 11 | 5 | Reference | | | 36 | 28 | Reference | | |
|  | absent | 3 | 2 | 0.682 | 0.085 - 5.448 | 0.718 | 16 | 19 | 0.655 | 0.286 - 1.5 | 0.317 |
| **GSTT1** | present | 10 | 6 | Reference | | | 38 | 40 | Reference | | |
|  | absent | 4 | 1 | 2.4 | 0.215 - 26.822 | 0.477 | 14 | 7 | 2.105 | 0.767 - 5.781 | 0.149 |
| **CAT (rs7943316)** | TT | 4 | 3 | Reference | | | 17 | 22 | Reference | | |
|  | TA | 10 | 3 | 0.4 | 0.055 - 2.886 | 0.363 | 29 | 18 | 0.48 | 0.202 - 1.138 | 0.096 |
|  | AA | 0 | 1 | 2.15E | 0 - | 1 | 6 | 7 | 0.902 | 0.256 - 3.181 | 0.872 |
| **TGF-**β**1 (rs1800469)** | CC | 9 | 4 | Reference | | | 21 | 19 | Reference | | |
|  | CT | 2 | 3 | 3.375 | 0.396 - 28.745 | 0.266 | 27 | 20 | 0.819 | 0.351 - 1.912 | 0.644 |
|  | TT | 3 | 0 | 0 | 0 - | 0.999 | 4 | 8 | 2.211 | 0.572 - 8.537 | 0.25 |
| **NQO1**  **(rs1131341)** | CC | 11 | 5 | Reference | | | 44 | 40 | Reference | | |
|  | CT | 3 | 2 | 1.467 | 0.184 - 11.718 | 0.718 | 6 | 6 | 1.1 | 0.328 - 3.688 | 0.877 |
|  | TT | 0 | 0 |  |  |  | 2 | 1 | 0.55 | 0.048 - 6.3 | 0.631 |
| **ATM**  **(rs3218698)** | TT | 11 | 7 | Reference | | | 45 | 42 | Reference | | |
|  | T/-T | 3 | 0 | 0 | 0 - | 0.999 | 7 | 5 | 0.765 | 0.225 - 2.598 | 0.668 |
| **RAD51 (rs1801321)** | GG | 13 | 4 |  | | | 32 | 26 | Reference | | |
|  | GT | 1 | 2 | 6.5 | 0.46 - 91.924 | 0.166 | 11 | 12 | 1.343 | 0.51 - 3.535 | 0.551 |
|  | TT | 0 | 1 | 5.25E | 0 - | 1 | 9 | 9 | 1.231 | 0.427 - 3.549 | 0.701 |
| **RAD51**  **(rs1801320)** | GG | 10 | 5 | Reference | | | 38 | 30 | Reference | | |
|  | CG | 4 | 2 | 1 | 0.134 - 7.451 | 1 | 13 | 15 | 1.462 | 0.604 - 3.536 | 0.4 |
|  | CC | 0 | 0 |  |  |  | 1 | 2 | 2.533 | 0.219 - 29.29 | 0.457 |
| **NBN (rs1805794)** | GG | 5 | 2 | Reference | | | 19 | 14 | Reference | | |
|  | CG | 8 | 2 | 0.625 | 0.065 - 5.966 | 0.683 | 28 | 21 | 1.018 | 0.417 - 2.485 | 0.969 |
|  | CC | 1 | 3 | 7.5 | 0.458 - 122.696 | 0.158 | 5 | 12 | 3.257 | 0.932 - 11.38 | 0.064 |
| **NBN**  **(rs1805787)** | GG | 11 | 5 | Reference | | | 33 | 35 | Reference | | |
|  | GC | 3 | 1 | 0.733 | 0.06 - 8.915 | 0.808 | 19 | 10 | 0.496 | 0.201 - 1.222 | 0.128 |
|  | CC | 0 | 1 | 3.55E | 0 - | 1 | 0 | 2 | 1.52E | 0 | 0.999 |
| **Ku70**  **(rs2267437)** | CC | 12 | 6 | Reference | | | 29 | 31 | Reference | | |
|  | CG | 1 | 1 | 2 | 0.106 - 37.83 | 0.644 | 18 | 12 | 0.624 | 0.256 - 1.517 | 0.298 |
|  | GG | 1 | 0 | 0 | 0 - | 1 | 5 | 4 | 0.748 | 0.183 - 3.062 | 0.687 |
| **Ku80**  **(rs3835)** | AA | 12 | 4 | Reference | | | 41 | 35 | Reference | | |
|  | GA | 2 | 3 | 4.5 | 0.542 - 37.378 | 0.164 | 9 | 11 | 1.432 | 0.532 - 3.852 | 0.477 |
|  | GG | 0 | 0 |  |  |  | 2 | 1 | 0.586 | 0.051 - 6.737 | 0.668 |
| **XRCC4**  **(rs1805377)** | GG | 11 | 7 | Reference | | | 33 | 33 | Reference | | |
|  | GA | 3 | 0 | 0 | 0 - | 0.999 | 17 | 11 | 0.647 | 0.263 - 1.59 | 0.343 |
|  | AA | 0 | 0 |  |  |  | 2 | 3 | 1.5 | 0.235 - 9.569 | 0.668 |
| **LIG4**  **(rs1805388)** | CC | 12 | 7 | Reference | | | 40 | 41 | Reference | | |
|  | CT | 2 | 0 | 0 | 0 - | 0.999 | 11 | 4 | 0.355 | 0.104 - 1.207 | 0.097 |
|  | TT | 0 | 0 |  |  |  | 1 | 2 | 1.951 | 0.17 - 22.379 | 0.591 |
| **SOD2**  **(rs4880)** | CC | 3 | 2 | Reference | | | 12 | 16 | Reference | | |
|  | CT | 8 | 4 | 0.75 | 0.087 - 6.468 | 0.794 | 27 | 20 | 0.556 | 0.216 - 1.431 | 0.223 |
|  | TT | 3 | 1 | 0.5 | 0.028 - 8.952 | 0.638 | 13 | 11 | 0.635 | 0.212 - 1.902 | 0.417 |
| **XRCC3 (rs861539)** | CC | 11 | 5 | Reference | | | 31 | 27 | Reference | | |
|  | CT | 3 | 2 | 1.467 | 0.184 - 11.718 | 0.718 | 19 | 20 | 1.209 | 0.536 - 2.724 | 0.648 |
|  | TT | 0 | 0 |  |  |  | 2 | 0 | 0 | 0 | 0.999 |

Supplementary table S2: Univariate analysis of candidate single nucleotide polymorphisms and radiation-induced oral mucositis after categorising the samples based on chemoradiotherapy and radiotherapy alone.
